# The Multi-Faceted Role of Autophagy During Animal Virus Infection

**DOI:** 10.3389/fcimb.2022.858953

**Published:** 2022-03-25

**Authors:** Hui Jiang, Xianjin Kan, Chan Ding, Yingjie Sun

**Affiliations:** ^1^ Department of Avian Infectious Diseases, Shanghai Veterinary Research Institute. Chinese Academy of Agricultural Science, Shanghai, China; ^2^ Jiangsu Co-innovation Center for Prevention and Control of Important Animal Infectious Diseases and Zoonosis, Yangzhou University, Yangzhou, China

**Keywords:** autophagy, animal virus, zoonotic diseases, porcine, avian

## Abstract

Autophagy is a process of degradation to maintain cellular homeostatic by lysosomes, which ensures cellular survival under various stress conditions, including nutrient deficiency, hypoxia, high temperature, and pathogenic infection. Xenophagy, a form of selective autophagy, serves as a defense mechanism against multiple intracellular pathogen types, such as viruses, bacteria, and parasites. Recent years have seen a growing list of animal viruses with autophagy machinery. Although the relationship between autophagy and human viruses has been widely summarized, little attention has been paid to the role of this cellular function in the veterinary field, especially today, with the growth of serious zoonotic diseases. The mechanisms of the same virus inducing autophagy in different species, or different viruses inducing autophagy in the same species have not been clarified. In this review, we examine the role of autophagy in important animal viral infectious diseases and discuss the regulation mechanisms of different animal viruses to provide a potential theoretical basis for therapeutic strategies, such as targets of new vaccine development or drugs, to improve industrial production in farming.

## Introduction to Autophagy

**Figure 1:**
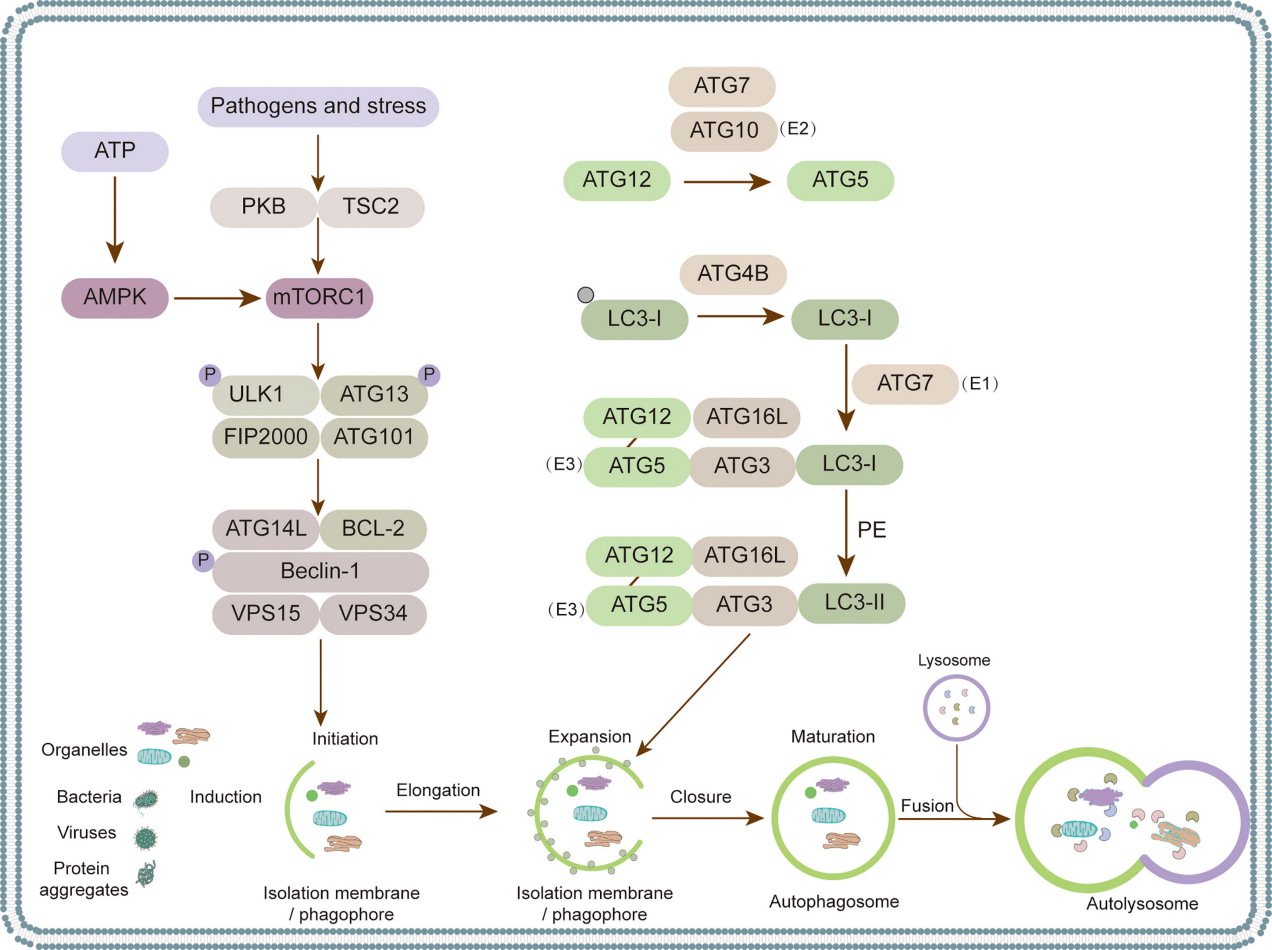
Autophagosome biogenesis and autophagy-related proteins. Five steps of autophagy process: (1) isolation membrane formation, (2) autophagic vesicle extension, (3) autophagosome maturation, (4) fusion of autophagosome and lysosome, (5) degradation of cargos. Various protein complexes function in multiple steps of autophagy. ULK1–ATG13–FIP200 complex is first recruited to the cargo sites and triggers the nucleation of isolation membranes. The second complex is phosphatidylinositol 3-phosphate (PtdIns3P), which consists of vps34, beclin-1 and Atg14, responsible for local production of PtdIns3P to recruit downstream effectors. The third complex is the Atg12-Atg5 conjugate system, catalyzed by E1 enzyme Atg7 and E2 enzyme Atg10, interacting with Atg16. The fourth complex is the one associated with ATG8 maturation, which catalyzes the binding of Atg8 to phosphatidylethanolamine PE through Atg7 and Atg3.

Autophagy is an important conserved cellular process that wraps damaged proteins, organelles, and microorganisms with autophagy vesicles, with a bilayer membrane structure for digestion by lysosomes (Parzych and Klionsky, 2014; Cong et al., 2020). Autophagy formation and maturation are highly complex processes that are strictly regulated by autophagy-related genes (ATGs) (Garcia and Shaw, 2017). The autophagy pathway is divided into five stages: initiation, expansion, maturation, fusion and degradation (**Figure 1**) (Yin et al., 2019). The mammalian target of rapamycin (mTOR) is a highly conserved protein kinase that senses various stresses, such as hunger, oxidative stress, energy stress, and pathogen infection, and initiates autophagy (Mayer et al., 2011; Garcia and Shaw, 2017). Various stress signals can inhibit mTOR and thus directly or indirectly activate autophagy (Yin et al., 2019). For example, adenosine 5’-monophosphate (AMP)-activated protein kinase (AMPK) senses cellular energy and ATP and thus activates autophagy by inhibiting mTOR complex 1 (mTORC1) (Mayer et al., 2011; Garcia and Shaw, 2017). Protein kinase B (PKB) is a negative regulator that senses pathogen infection or stress, followed by the phosphorylation of tuberous sclerosis complex 2 (TSC2) and the activation of mTORC1, thus inhibiting autophagy (Zalckvar et al., 2009; Dan et al., 2014). The inhibition of mTORC1 activates ULK1/2 complexes composed of ULK1 or ULK2 kinases, ATG13, FIP2000, and ATG101 (Hosokawa et al., 2009; Chen and Klionsky, 2011). In addition to the mTORC1 pathway, there are various mTORC1-independent pathways, such as the endoplasmic reticulum stress (ERS) pathway and the inositol phospholipid signal pathway (Sarkar, 2013). After autophagy initiation, the membrane expands and nucleates, forming bilayer isolation membranes and phagophores. This process is mainly mediated by a class III phosphatidylinositol 3-kinase (PI3K) complex composed of Vps34, Vps15, and beclin-1 (Becn1) (He and Klionsky, 2009). Beclin-1 combines with Vps34 to promote membrane nucleation (Liang et al., 2008). The phosphatidylinositol-3-phosphate (PI3P) gathered at the membrane nucleation site needs to recruit more ATGs to start autophagosome extension and membrane closure (Yin et al., 2019). This process requires two ubiquitin-like conjugation systems: the ATG12-ATG5-ATG16L1 system and the LC3 system (Mizushima et al., 2011; Shpilka et al., 2011). ATG12 and ATG5 form covalent conjugations under the catalysis of ATG7 and ATG10, thus further recruiting ATG16L1 to form the ATG12-ATG5-ATG16L1 complex with an E3-like function (Mizushima et al., 2011; Shpilka et al., 2011; Tsuboyama et al., 2016). The LC3 system involves the binding of LC3 and phosphatidylethanolamine (PE) (Yang et al., 2009). LC3-I, the precursor of LC3, exposes glycine residues at its carboxyl terminal under ATG4 splicing, which combines with PE to form LC3-II (Nakatogawa et al., 2007) (**Figure 1**). Autophagy is a dynamic process, and autophagic flux—in which the rate of autolysosomes degrades autophagy substrates—is often used to judge the stage of autophagy (Loos et al., 2014; Galluzzi et al., 2017). Chloroquine (CQ), bafilomycin A1 (Baf A1), and 3-methyladenine (3-MA) are autophagy inhibitors. Chloroquine and bafilomycin A1 are used to detect autophagy flux by inhibiting the fusion of autophagosomes and lysosomes (Mauthe et al., 2018). CQ also inhibits the degradation of cargos by autolysosomes, whereas 3-MA inhibits the formation of autophagosomes through inhibiting PI3K (Wu et al., 2013). Rapamycin is a widely used autophagy activator that specifically inhibits mTOR (Li et al., 2014a).

Traditionally, autophagy is considered a process in which cells nonselectively degrade intracellular substances under the stimulation of starvation. However, there is a great deal of evidence showing that autophagy can specifically degrade aggregated proteins, damaged cellular organelles (mitochondria, peroxisomes, endoplasmic reticulum (ER), nucleus, lysosomes, lipid droplets, Golgi apparatus, and ribosomes), and pathogens through autophagy receptors (Shaid et al., 2013; Stolz et al., 2014; Gatica et al., 2018). The autophagy receptor is a bridge between the ubiquitin substrate and light chain LC3 on the inner membrane of autophagosomes (Shaid et al., 2013). Selective autophagy requires the participation of autophagy receptors. At present, the known selective autophagy receptors are SQSTM1 (sequestosome 1, also known as p62), NBR1 (neighbor of BRCA1 gene 1), CALCOCO2 (calcium binding and coiled-coil domain 2, also known as NDP52), RETREG1 (Reticulophagy Regulator 1, also known as FAM134B), LGALS3 (galectin 3), and OPTN (optineurin) (Lamark et al., 2009; Deosaran et al., 2013; Maejima et al., 2013; Khaminets et al., 2015; Lazarou et al., 2015). RETREG1/FAM134B is a specific receptor of reticulophagy that selectively degrades ER (Khaminets et al., 2015). NBR1 and p62 participate in pexophagy, which selectively degrades peroxisomes (Deosaran et al., 2013; Gatica et al., 2018). NDP52 and OPTN are involved in xenophagy (Wild et al., 2011), which is the selective degradation of intracellular pathogens, including bacteria, fungi, parasites, and viruses (Colombo et al., 2006; Gomes and Dikic, 2014). The process of selective degradation of viruses by autophagy is also called virophagy (Galluzzi et al., 2017).

**Figure 2:**
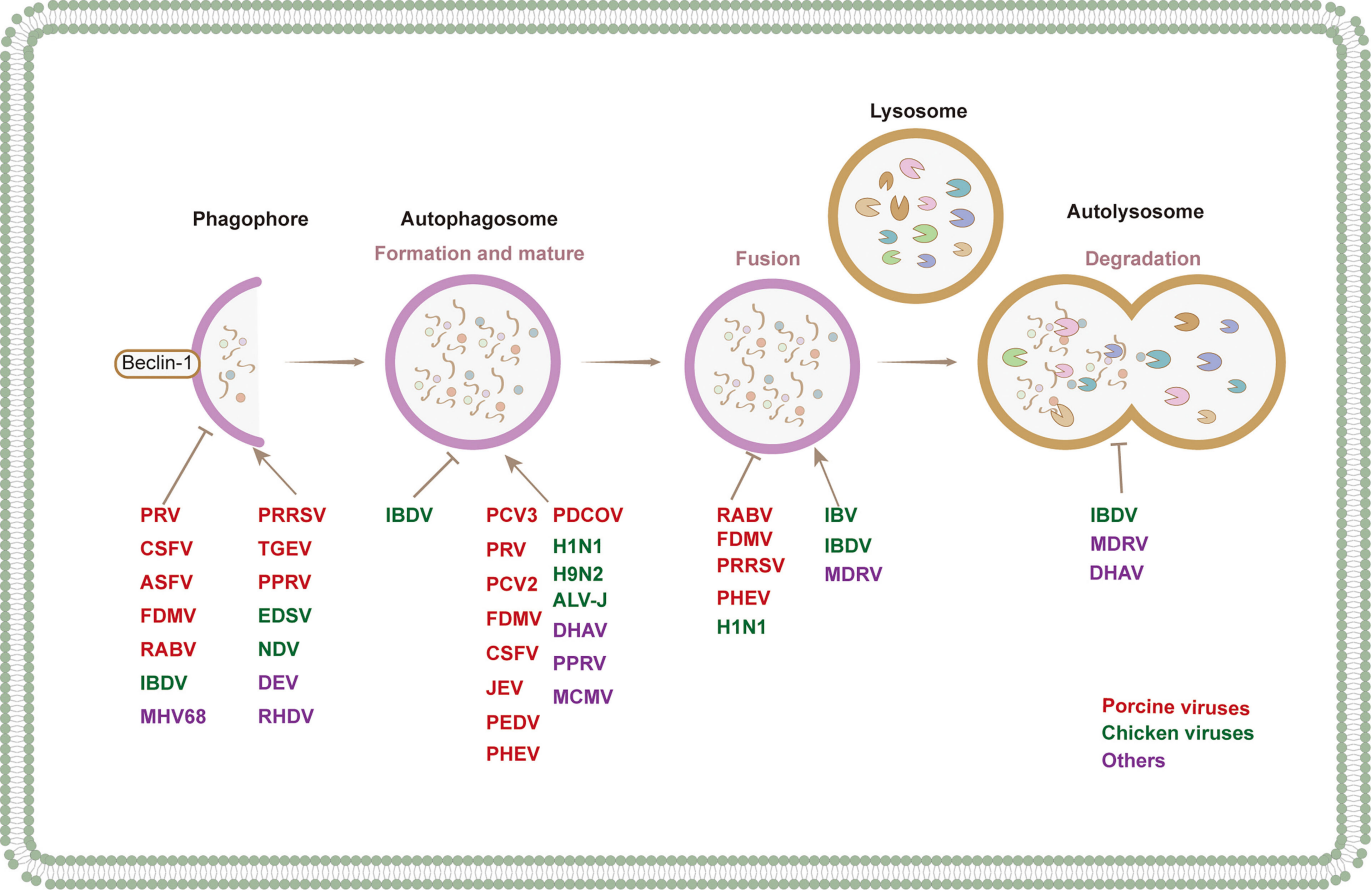
The modulation of autophagy at different stages by different species of animal viruses. Red, blue and purple fonts refer to porcine virus, chicken virus and others, respectively.

Growing evidence has confirmed the interaction between animal viruses and autophagy (**Figure 2**). The consequences of virus-autophagy interaction can be divided into the following three categories: (1) Autophagy activation promotes virus replication; for example, the Newcastle disease virus (NDV) exploits autophagy to promote virus replication (Meng et al., 2012a). (2) Autophagy plays a negative role in virus replication, such as the Japanese encephalitis virus (JEV) (Sharma et al., 2014). (3) Autophagy has no effect on virus infection; for example, equine herpesvirus 1 (EHV-1) activates autophagy, which has no effect on its replication (Cymerys et al., 2014) (**Figure 2**). Over the past few decades, researchers have focused mainly on the interaction between human viruses and autophagy, while relatively few reports have reviewed the interactions between animal viruses and autophagy. In this review, we summarize the interactions between different animal viruses and their hosts and examine how the virus destroys or exploits autophagy to play a role in affecting the pathogenesis of the virus. This review provides a theoretical basis for the clinical research and development of viral therapeutic drugs or vaccines.

## Current Knowledge of Autophagy in Different Animal Viruses

**Figure 3:**
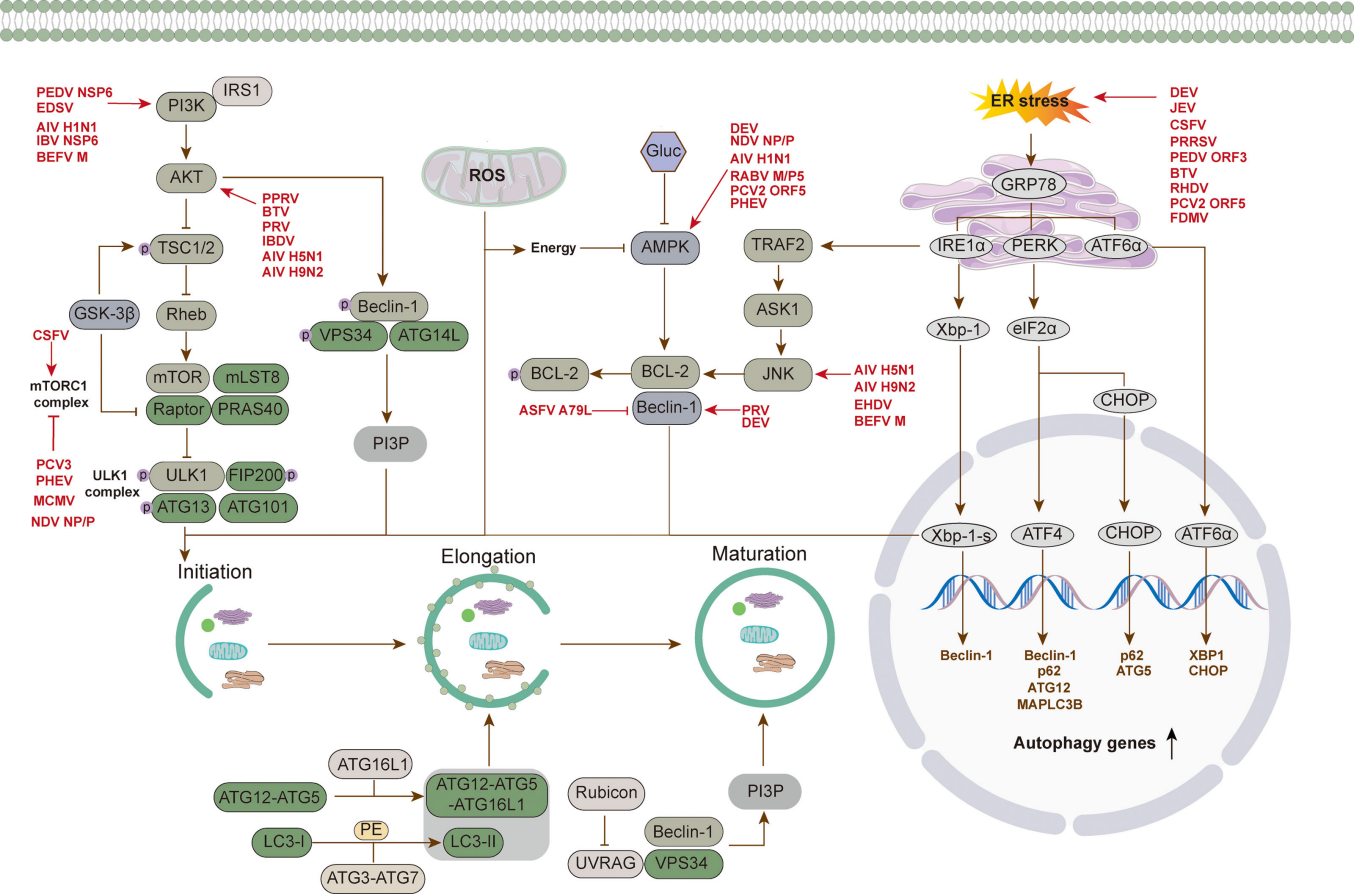
Regulation mechanism of autophagy *via* different signal pathways by animal virus. Animal viruses regulate the process of autophagy through different signaling pathways: (1) the regulation of canonical PI3K-AKT-mTOR and AMPK-mTOR-ULK1 signaling pathways to promote the initiation of autophagy. (2) regulation of pathways involved in ER stress response, such as IRE1α-XBP1 and PERK-eIF2α-ATF4 signaling pathways. (3) regulation of pathways involved in oxidative stress, such as ROS generation by mitochondrial β oxidation regulating the occurrence of autophagy through downstream pathways.

Numerous studies have shown the manipulation of autophagy by animal viruses. In the following chapters, we summarize the interactions between autophagy and porcine, avian, ruminant, and other animal viruses (**Table 1** and **Figure 3**).

**Table 1:** Summary of known interactions between animal viruses and autophagy.

Host	Virus	Effect (s) of Autophagy on Virus	Mechanism of Virus-Autophagy Interaction	Reference
Human, Pig, Cat, Dog and so on	Rabies virus (RABV)	Increases RABV replication	RABV induces complete autophagy in SK cells, but incomplete autophagy in NA cells	(Peng et al., 2016; Liu et al., 2017; Liu et al., 2020)
			P5 binds to beclin-1 to induce incomplete autophagy through CASP2-AMPK-MAPK and CASP2-AMPK-AKT-MTOR pathways	
Pig, Bovine, sheep, Musk deer and so on	Foot-and-mouth disease virus (FMDV)	Increases FMDV infection *in vitro* and *in vivo*	VP2 interacts with HSPB1 to activate autophagy through EIF2S1-ATF4 pathway	(O’Donnell et al., 2011; Liao et al., 2013; Sun et al., 2018)
			2C activates autophagy depended on WIPI1, WIPI2, ATG5 and ATG7	
Pig	African swine fever virus (ASFV)	Inhibits ASFV replication	E199L downregulated PYCR2 to induce complete autophagy	(Berryman et al., 2012; Hernaez et al., 2013; Chen et al., 2021)
			A179L interacts with beclin-1to inhibit autophagy	
Pig	Pseudorabies virus (PRV)	Increases PRV replication in N2a cells	US3 inhibits autophagy through AKT/mTOR pathway	(Sun et al., 2017; Xu et al., 2018)
		Inhibits PRV replication in PK-15 cells.	PRV infection in N2a cells activates autophagy through beclin-1-ATG7-ATG5 pathway	
Pig	Porcine parvovirus (PPV)	Increases PPV infection	PPV exploits MAPKs (p38 and ERK1/2), PKC and Ca^2+^ to induce incomplete autophagy	(Zhang et al., 2019b; Zhao et al., 2021)
Pig	Porcine circoviruses (PCVs)	Increases PCV2 replication	PCV2 activates autophagy through activating AMPK and increasing host oxidative stress	(Gu et al., 2016; Qian et al., 2017; Geng et al., 2020; Guo et al., 2020; Han et al., 2020; Lv et al., 2020; Zhang et al., 2020c)
			PCV2 induces mitophagy by increasing ROS production and the phosphorylation of Drp1	
			ORF5 activates autophagy through AMPK-ERK1/2-mTOR and PERK-eIF2 α-ATF4 pathways	
			ORF5 inhibits autophagy by binding to YWHAB	
			Cap activates autophagy through binding to pDNAJB6	
			Cap induces complete autophagy *via* inhibition of p-mTOR	
Pig	Classical swine fever virus (CSFV)	Increases CSFV replication	CSFV induces autophagy *via* activating PERK and IRE1 pathways	(Pei et al., 2014; Gou et al., 2017; Luo et al., 2018; Fan et al., 2021; Zhang et al., 2021a; Zhu et al., 2021)
			CSFV induces autophagy by down-regulation of ROS-dependent RLR signals	
			NS5A binds to LC3 to activate autophagy	
			NS3 binds to LDHB to induce mitophagy	
Pig	Japanese encephalitis virus (JEV)	Promotes JEV replication in human NT-2 cells	JEV activates autophagy through ERS induced by XBP1 and ATF6 in Neuro2a cells	(Li et al., 2012; Wang et al., 2015b; Sharma et al., 2017; Xu et al., 2021)
		Inhibits JEV replication in Neuro2a cells	NS1 and vRNA colocalize with LC3 to activate autophagy	
			NS3 targets IRGM to activate autophagy	
Pig	Porcine reproductive and respiratory syndrome virus (PRRSV)	Increases PRRSV replication	PPRSV activates autophagy through ERS induced by PERK and IRE1 pathway	(Sun et al., 2012a; Wang et al., 2019a; Cao et al., 2020; Chen et al., 2020)
			PRRSV induces lipophagy by down-regulation of NDRG	
			NSP2 binds to 14-3-3ϵ to induce aggrephagy	
			NSP2 colocalizes with LC3 and activates incomplete autophagy	
Pig	Transmissible Gastroenteritis Virus (TGEV)	Inhibits TGEV replication in ST cells and PK15 cells	Unknown	
		Increases virus replication in IPEC-J2 cells		
Pig	Porcine Epidemic Diarrhea Virus (PEDV)	Increases PEDV infection in IPEC-J2 cells	PEDV induces autophagy *via* PERK and IER1 pathway	(Zou et al., 2019; Lin et al., 2020; Sun et al., 2021)
			Nsp6 activates PI3K/Akt/mTOR pathway	
		Inhibits PEDV replication in IECs, Vero and LLC-PK1 cells	ORF3 activates autophagy through PERK-eIF2a pathway	
Pig	Porcine hemagglutinating encephalomyelitis virus (PHEV)	Facilitates PHEV replication	PHEV activates autophagy independent of the classic AMPK-mTORC1-ULK1 pathway	
Pig	Porcine deltacoronavirus (PDCOV)	Increases PDCOV replication	PDCOV activates autophagy through p38 signal pathway	(Duan et al., 2021)
Human, Pig, Horse, Poultry and so on	Influenza A viruses (IAVs)	Increases H1N1 replication in MEFs	H1N1 activates autophagy through PI3K pathway	(Ma et al., 2011; Zhirnov and Klenk, 2013; Wang et al., 2019b; Yu et al., 2019; Zhang et al., 2019a; Zhao et al., 2020; Zhang et al., 2021b)
		Inhibits H1N1 replication in A549 cells.	H1N1 activates autophagy through AMPKα-ULK1 pathway	
		Increases	H1N1 inhibits autophagy by promoting the formation of Circ-GATAD2A in A549 cells	
		H5N1 replication	H5N1 activates autophagy through AKT- mTOR or TSC2 pathway H5N1 activates autophagy *via* activating JNK and inhibiting PI3K pathway	
		Increases	H9N2 induces autophagy by activating Akt/TSC2/mTOR pathway and PI3K/JNK pathway	
		H9N2 replication	PB2, NP and M2 encoded by H5N1 activates autophagy through AKT-mTOR pathway	
Chicken	Egg drop syndrome virus (EDSV)	Increases EDSV replication	EDSV activates autophagy *via* PI3K/Akt/mTOR pathway	(Wang et al., 2018)
Chicken	Newcastle disease virus (NDV)	Increases NDV replication	NDV induces mitophagy	(Sun et al., 2014; Bu et al., 2016; Cheng et al., 2016; Ren et al., 2019);
			HN and F protein cooperately activates autophagy through AMPK/mTORC/ULK1 pathway	
			NP and P protein activates autophagy through inducing ERS	
Chicken	Infectious bronchitis virus (IBV)	Unknown	Nsp6 activates autophagy by activation of PI3K	(Cottam et al., 2011; Cottam et al., 2014)
Chicken	Infectious bursal disease virus (IBDV)	Promotes IBDV maturation and release	VP2 binds to HSP90AA1 to trigger autophagy through AKT-mTORC pathway	(Hu et al., 2015; Zhang et al., 2020b)
			VP3 inhibits autophagy through destroying PIK3C3-beclin-1 complex and PIK3C3-PDPK1 complex	
Chicken	Avian reovirus (ARV)	Increases ARV replication	ARV activates autophagy through PI3K/Akt/mTOR pathway	(Meng et al., 2012b; Chi et al., 2013; Duan et al., 2015; Li et al., 2015; Huang et al., 2017)
			P17 activates autophagy through activation of PTEN, AMPK and PKR/eIF2 pathways	
			p17 mediated inhibition of Akt leads to activation of autophagy	
			σA and σNS plays roles in activation of incomplete autophagy	
Chicken	Avian leukosis virus subgroup J (ALV-J)	Inhibits ALV-J replication	ALV-J inhibits autophagy through GADD45β/MEKK4/p38MAPK pathway	(Lu et al., 2013; Liao et al., 2020)
Duck	Duck enteritis virus (DEV)	Promotes DEV propagation	DEV triggers autophagy through activating ERS mediated by the activation of PERK and IRE1 pathways	(Yin et al., 2017a; Yin et al., 2017b; Yin et al., 2017c; Yin et al., 2018; Wu et al., 2019)
			DEV induces autophagy through the activation of AMPK-TSC2-MTOR pathway and CaMKK β-AMPK	
			DEV downregulates miR-30a-5p and increases beclin-1-mediated autophagy	
Duck	Muscovy duck reovirus (MDRV)	Promotes MDRV replication	Unknown	
Duck	Duck hepatitis A virus (DHAV)	Enhances DHAV replication	Unknown	
Sheep	Bluetongue virus (BTV)	Increases BTV replication	BTV activates autophagy by the inhibition of AKT-TSC2-mTOR pathway and the up-regulation of AMPK-TSC2-mTOR pathway	(Lv et al., 2015a; Lv et al., 2015b; Lv et al., 2016a; Lv et al., 2016b)
			BTV activates autophagy by ERS mediated by PERK-eIF2α pathway	
			BTV activates autophagy by destroying cell energy metabolism	
Sheep	Peste des petits ruminants virus (PPRV)	Facilitates PPRV replication	H induces autophagy through inhibition of AKT-MTOR pathway	(Yang et al., 2018; Yang et al., 2020a)
			C and N induces autophagy by binding to IRGM and HSPA1A	
Sheep	Caprine parainfluenza viruses type 3 (CPIV3)	Inhibits CPIV3 replication	CPIV3 inhibits autophagy mediated by exosomes	(Mao et al., 2020)
Bovine	Epizootic Hemorrhagic Disease Virus (EHDV)	Increases EHDV replication	EHDV activates autophagy by JNK pathway	(Shai et al., 2013)
Bovine	Bovine viral diarrhea virus (BVDV)	Facilitates BVDV propagation	Unknown	
Bovine	Bovine Epidemic Fever Virus (BEFV)	Increases BEFV replication	BEFV triggers autophagy *via* PI3K/Akt/NF-κB and Src/JNK/AP1 pathway	(Cheng et al., 2019; Tseng et al., 2020)
			M activates autophagy through inhibition of PI3K/Akt/mTOR pathway	
Monkey	Rhesus monkey rhadinovirus (RRV)	Maintains cell survival	Unknown	
Hourse	Equine herpesvirus 1(EHV-1)	No effect on EHV-1 replication	Unknown	
Rabbit	Rabbit Hemorrhagic Disease Virus (RHDV)	promotes RHDV replication	At the early stage of infection, RHDV rapidly activates autophagy by induced ERS; At the late infection, RHDV promotes apoptosis to inhibit autophagy	(Vallejo et al., 2014)
Mouse	Murine cytomegalovirus (MCMV)	Increases MCMV replication	MCMV activates autophagy through mTOR signal pathway	
Mouse	Murine gammaherpesvirus 68 (MHV68)	Promotes MHV68 reactivation	M11 binds beclin-1 to inhibit autophagy	(Ku et al., 2008; Su et al., 2014)

### The Role of Autophagy in Viruses Causing Pig Diseases

#### Porcine DNA Viruses

African swine fever virus (ASFV) is a member of the large DNA virus family and belongs to the family *Asfarviridae* (Netherton et al., 2019). The genome is double-stranded linear DNA, encoding more than 150 proteins (Chen et al., 2021). ASFV infection causes a highly contagious and fatal hemorrhagic fever in pigs (Costard et al., 2013). However, there are few studies on autophagy caused by ASFV infection. Hernaez et al. (2013) discovered that the viral protein A179L encoded by ASFV, which is homologous to B-cell lymphoma-2 (Bcl2), interacts with the autophagy regulatory factor beclin-1 to inhibit autophagy (Hernaez et al., 2013). A further study showed that, similar to Bcl-2, A179L inhibits autophagy by binding to beclin-1 through its BH3 binding grooves (Banjara et al., 2019). Moreover, E199L is a viral inner membrane protein necessary for virus entry (Matamoros et al., 2020). E199L interacts with PYCR2 and downregulates the expression of PYCR2, and therefore activates complete autophagy (Chen et al., 2021).

Pseudorabies virus (PRV), also known as Suid herpesvirus 1 (suHV-1), is a double-stranded DNA virus, which belongs to the subfamily *α herpesvirus* (Muller et al., 2011; Yu et al., 2014). The virus has a wide range of infected hosts, and pigs are the only natural reservoir hosts. PRV causes Aujeszky disease in adult pigs, leading to significant global economic losses (Pomeranz et al., 2005; Sun et al., 2017). The role of autophagy in human herpesvirus has been widely studied. For example, Herpes simplex virus 1 (HSV-1) ICP34.5 binds to autophagy regulatory gene beclin-1, thus inhibiting autophagy (Orvedahl et al., 2007). However, there are few studies on autophagy in PRV. Sun et al. (2017) confirmed the relationship between PRV and autophagy. Their research found that high MOI PRV could activate autophagy without virus replication at the early stage of infection, while with virus replication, the viral envelope protein US3 reduced the level of autophagy by activating the AKT/mTOR pathway to promote virus replication (Sun et al., 2017). By contrast, another report showed that PRV infection of mouse neuro-2a (N2a) cells significantly increased the transformation of LC3-I to LC3-II and the number of autophagosomes (Xu et al., 2018). In addition, the autophagy inducer rapamycin promotes virus replication, while the autophagy inhibitor 3-MA inhibits virus replication, indicating that PRV activates autophagy through the beclin-1-ATG7-ATG5 pathway, thus promoting virus replication (Xu et al., 2018). It was found to vary PRV strains induced the different level of autophagy (Xu et al., 2018). The level of autophagy induced by mutant ZJ01 infection was higher than that of the LA vaccine strain (Xu et al., 2018). In short, whether autophagy is activated or inhibited by PRV infection relates to the type of infected cells and the virus strains.

Porcine parvovirus (PPV) is one of the main pathogens causing reproductive disorders in sow (Cotmore et al., 2014; Zhang et al., 2019c). It belongs to the genus *Protoparvovirus* of the family *Parvoviridae* (Mengeling and Cutlip, 1976). The viral genome is a single-stranded linear DNA, encoding four nonstructural proteins (NS1, NS2, NS3, and NS4) and three structural proteins (VP1, VP2, and VP3) (Bergeron et al., 1996; Simpson et al., 2002; Zadori et al., 2005). At present, few studies have investigated the interaction between PPV and autophagy. Zhang et al. (2019c) reported that PPV infection in porcine placental trophoblasts cells can induce autophagy, which promotes virus replication. PPV exploits MAPKs (p38 and ERK1/2), protein kinase C (PKC), and Ca^2+^ to induce incomplete autophagy (Zhao et al., 2021).

Porcine circoviruses (PCVs) are members of the genus *circovirus* in the family *Cycloviridae* (Ellis, 2014). The genome of PCV is a single-stranded circular DNA of 1.76 kb. It is, by far, the smallest DNA virus found to infect mammals (Delwart and Li, 2012). There are three types of PCVs: PCV1 (Tischer et al., 1974), PCV2 (Allan et al., 1998), and PCV3 (Phan et al., 2016; Palinski et al., 2017). PCV1 is not pathogenic to pigs and is a contaminant in PK-15 cells (Tischer et al., 1974). PCV2 is a pathogen of porcine circovirus-associated diseases that causes significant economic losses (Ellis, 2014). PCV3 is a new virus found in some pig-raising countries in recent years (Phan et al., 2016; Faccini et al., 2017; Kwon et al., 2017; Franzo et al., 2018; Hayashi et al., 2018; Kedkovid et al., 2018). PCV3 infection induces complete autophagy by inhibiting the phosphorylation of mTORC. Cap protein encoding by PCV3 induces autophagy (Geng et al., 2020). Accumulating evidence has revealed an interaction between PCV2 and autophagy. Zhu et al. (2012) found that PCV2 infection in PK-15 cells induces autophagosome formation, increases autophagy flux, and promotes virus replication. Mechanismly, PCV2 upregulates calcium/calmodulin-dependent protein kinase (CaMK) kinase β (CaMKKβ) by increasing cytoplasmic Ca^2+^ through inositol 1,4,5-triphosphate receptors (IP3R) (Gu et al., 2016). CaMKKβ activates both AMPK and CaMKI, while the activation of AMPK phosphorylates TSC2, thus activating autophagy by mTORC inhibition. The activation of autophagy, in turn, promotes virus replication (Zhu et al., 2012; Gu et al., 2016; Ren et al., 2016). Except for activating autophagy through this pathway, PCV2 activates autophagy by increasing host oxidative stress and inhibiting apoptosis (Qian et al., 2017; Zhai et al., 2019). The inhibition of reactive oxygen species (ROS) production by taurine, selenizing astragalus polysaccharide, and SeMet inhibits autophagy and PCV2 replication (Liu et al., 2018; Qian et al., 2018; Zhai et al., 2018; Zhai et al., 2019). Several PCV2 proteins are involved in autophagy. ORF5 activates autophagy through AMPK-ERK1/2-mTOR and PERK-eIF2α-ATF4 pathways, thus promoting PCV2 replication (Lv et al., 2020). However, another study showed that ORF5 directly binds to host regulatory factor 14-3-3β (YWHAB), which inhibits ERS, ROS, and autophagy, thus inhibiting virus replication (Guo et al., 2020). In addition, PCV2 infection increases the expression of pDNAJB6 in PK-15 cells, and the C-terminal J domain of pDNAJB6 binds to the cap protein, which increases the formation of autophagy and promotes virus replication (Han et al., 2020). Upon PCV2 infection, host microRNAs play an important role in regulating autophagy. PCV2 upregulates miR-30a-5p, which directly targets the 14-3-3 gene, leading to cell cycle arrest at the G2 phase, and thus promoting autophagy and triggering PCV2 replication (Wang et al., 2017b). PCV2 infection induces not only autophagy but also mitophagy. PCV2 infection increases the production of ROS and the phosphorylation of dynamin-related protein 1 (Drp1), upregulates the expression of PTEN-induced kinase 1 (PINK1), and stimulates the recruitment of Parkin to mitochondria, thus inducing mitophagy (Zhang et al., 2020c).

#### Porcine RNA Viruses

Rabies virus (RABV) is a neurophagic virus that can infect people and animals and cause fatal rabies (Li et al., 2017). It is a member of the *Lyssavirus* genus of the *Rhabdoviridae* family (Tu et al., 2018). The genome is a single negative-strand RNA that encodes five viral proteins (Banerjee et al., 1991). Peng et al. (2016) reported that both wild-type (WT) and attenuated strains can trigger autophagy, but compared with attenuated strains, WT strains have a stronger ability to induce autophagy. Notably, the level of LC3-II/LC3-I was upregulated upon RABV infection in both human neuroblastoma cells (SK) and mouse neuroblastoma cells (NA), but whereas SK cells showed complete autophagy, NA cells showed incomplete autophagy (Peng et al., 2016). In addition, the M protein encoded by RABV can cooperate with the virus to increase the occurrence of autophagy (Peng et al., 2016). Except for the role of M protein in autophagy, it was found that the 173-222 amino acid (aa) residues of the P5 protein directly bind to the N-terminal 1-139 residues of beclin-1, thus inducing incomplete autophagy through the activation of CASP2-AMPK-MAPK and CASP2-AMPK-AKT-MTOR pathways and providing a scaffold for virus replication (Liu et al., 2017; Liu et al., 2020). In addition, RABV induced autophagy in BV2 cells in a dose-dependent manner (Wang et al., 2021a).

Foot-and-mouth disease virus (FMDV) is an acute infectious infection virus that can cause more than 70 kinds of foot-and-mouth disease in animals (Grubman and Baxt, 2004; Jamal and Belsham, 2013). FMDV belongs to the *Aphthovirus* genus of the *Picornaviridae* family (Mason et al., 2003; Klein, 2009; Tulloch et al., 2014). The genome is a single positive-stranded RNA that can encode four structural proteins and eight nonstructural proteins (Logan et al., 2018). The interaction between FMDV and autophagy has been widely studied. PK15 cells infected with FMDV can rapidly induce LC3 lipidation and GFP-LC3 subcellular redistribution in the early stage, which has been proven to depend on ATG5 in mouse embryonic fibroblasts (Fan et al., 2017). In ATG5-deficient cells, the titer of FMDV decreased (Berryman et al., 2012). Treatment of FMDV-infected PK-15 cells with rapamycin increases the virus titer, while 3-MA inhibits virus replication, indicating that autophagy benefits virus replication (Sun et al., 2018). Autophagy induced by FMDV infection in MEFs is independent of Vps34’s class III PI3K activity because the PI3K inhibitor wortmannin treatment does not affect autophagosome formation (Sun et al., 2018). FMDV can also promote virus replication by activating the stimulator of interferon response cGAMP interactor 1 (STING1) to induce reticulophagy (Zhang et al., 2021b). In addition, accumulating studies have shown that viral proteins encoded by FMDV are involved in autophagy, thus regulating virus replication. Several FMDV viral proteins have been shown to be localized with autophagy-related proteins, such as nonstructural proteins 2B, 2C, and 3A localized with LC3, structural protein VP1 localized with ATG5, LC3, and lysosomal associated membrane protein 1 (LAMP-1) (O’Donnell et al., 2011). The FMDV capsid protein VP2 interacts with the small heat shock protein beta-1 (HSPB1) to activate autophagy through the eukaryotic translation initiation factor 2 subunit alpha (EIF2S1)-activating transcription factor 4 (ATF4) pathway, thus promoting virus replication (Sun et al., 2018). Moreover, UV-FMDV can also induce autophagy through the EIF2S1-ATF4 and AKT-MTOR pathways, indicating that FMDV-induced autophagy is independent of virus replication (Sun et al., 2018). FMDV infection not only activates but also inhibits autophagy to promote replication. In the process of FMDV infection, viral protein 2C binds to beclin-1, thus preventing the fusion of lysosomes and autophagosomes, and resulting in the survival of the virus (Gladue et al., 2012). In macrophages, the 2 C protein activates autophagy depending on WIPI1, WIPI2, ATG5, and ATG7, but not beclin-1 (Liao et al., 2013). Some controversial studies have also reported that autophagy is induced by FMDV infection through the PERK-eIF2α and ATF6 signaling pathways but has no effect on virus replication (Wu et al., 2021). The Seneca valley virus (SVV), another member of the *Picornaviridae* family, causes neonatal death and vesicular lesions in pigs (Hales et al., 2008). SVV infection also activates autophagy through the PERK and ATF6 unfolded protein reaction (UPR) pathways, thus promoting virus replication (Hou et al., 2019).

Classical swine fever virus (CSFV) is the pathogen of classical swine fever (Kleiboeker, 2002). It is a member of the *Pestivirus* genus within the *Flaviviridae* family, and its genome is a single positive-stranded RNA (Becher et al., 2003). CSFV infection causes high fever, multiple hemorrhages, nervous system diseases, and respiratory and gastrointestinal diseases in pigs (Kleiboeker, 2002; Lohse et al., 2012). Growing studies have demonstrated that CSFV activates autophagy to promote virus replication. Pei et al. (2014) discovered that CSFV induced the transformation of LC3-I/LC3-II and the level of the ATG5-ATG12 conjunction system, as well as the formation of autophagosomes. Interfering autophagy regulatory factors inhibits the production of offspring virus (Pei et al., 2014). Subsequently, Zhu et al. (2021) proved that CSFV-infected PK-15 and 3D4/2 cells activated ERS by activating the PERK and IRE1 pathways, thus inducing autophagy to promote virus replication. Gou et al. (2017a) demonstrated that CSFV infection activates the autophagy of splenocytes *in vivo* for the first time, and 3-MA inhibits CSFV replication *in vitro* and *in vivo*. In addition, CSFV infection activates the PINK1 and Parkin pathways to degrade mitofusin (MFN) 2, silence mitochondrial ATGs, and reduce virus titers, indicating that CSFV promotes virus replication by activating mitochondrial autophagy (Gou et al., 2017). Infectious time seems to be an important factor affecting CSFV-autophagy interaction. At the early stage of infection, CSFV infection inhibits autophagy by reducing the phosphorylation of the Akt/mTORC1/S6 pathway. At the late stage of infection, the CSFV activates mTORC1 to inhibit autophagy, which is conducive to the dynamic balance of cell survival and virus replication (Luo et al., 2018). Several nonstructural proteins encoded by CSFV can regulate autophagy. For example, by binding to LC3, the NS5A protein increases the expression of ATGs and activates complete autophagy, thus promoting virus replication. Notably, the phosphorylation of NS5A at 81 and 92 aa within its N-terminal region is essential for activating autophagy (Zhang et al., 2021a). Further, NS3 directly binds to lactate dehydrogenase B (LDHB), a key glycolysis metabolic enzyme that catalyzes the transformation of pyruvate and lactic acid in the anaerobic glycolysis pathway and inhibits LDHB, activating mitochondrial autophagy (Fan et al., 2021).

JEV is a mosquito-borne virus belonging to the family *Flaviridae* and genus *flavivirus* (Sharma et al., 2021), and it is the main cause of viral encephalitis (Sharma et al., 2021). The genome is a positive single-stranded RNA that encodes three structural proteins [nucleocapsid (C), membrane protein (M), and envelope protein (E)] and seven nonstructural proteins (NS1, NS2A, NS2B, NS3, NS4A, NS4B, and NS5) (Yun and Lee, 2018). Three different JEV-autophagy interplay models have been reported so far. In the first model, JEV infection activates autophagy to promote virus replication. Li et al. (2012) discovered that JEV virions can activate autophagy to promote virus replication. Autophagy is associated with the early infection steps of JEV infection. NS1 is colocalized with nonlipidated LC3 to influence the replication of JEV. Further analysis indicated that the viral replication is associated with ER-associated degradation (ERAD) pathway (Sharma et al., 2014). JEV C, M, and NS3 proteins can also activate autophagy. Among them, NS3 targets immunity-related GTPase M (IRGM) to activate autophagy and promote virus replication (Wang et al., 2015b). In the second model, JEV infection inhibits autophagy and promotes virus replication. JEV infection upregulates NEDD4, such as E3 ubiquitin protein ligase (Nedd4), in SK-N-SH neuroblastoma cells, thereby inhibiting autophagy and promoting virus replication (Xu et al., 2017). In the third model, JEV infection activates autophagy to inhibit virus replication. JEV infection in Neuro2a cells activates autophagy through ERS induced by XBP1 and ATF6. The depletion of XBP1 and ATF6 inhibits autophagy and increases JEV-infected cell death (Sharma et al., 2017; Sharma et al., 2018).

Porcine reproductive and respiratory syndrome virus (PRRSV) is a pathogen that causes severe respiratory distress and high mortality in piglets and reproductive failure in sows (Rossow, 1998). PRRSV belongs to the genus *Arterivirus* of the *Arteriviridae* family of order *Nidovirales*, and its genome is a single positive-stranded RNA (Cavanagh, 1997; Ziebuhr et al., 2000). An increasing number of studies have demonstrated that PRRSV infection can activate autophagy (Liu et al., 2012). PRRSV infection increases the transformation of LC3-I to LC3-II and double-membrane vesicles; 3-MA and ATG7-/beclin-1 knockdown inhibit virus replication; and rapamycin treatment induces autophagy to increase virus replication (Chen et al., 2012; Liu et al., 2012). (Wang et al., 2015a) subsequently confirmed *in vivo* that HP-PRRSV activates autophagy in thymocytes and bystander cells. PPRSV infection activates ERS through the PERK and IRE1 pathways to induce autophagy, and ERS and autophagy promote virus replication (Chen et al., 2020). Several viral proteins have been shown to be involved in autophagy activation. Upon PRRSV infection of Marc145 cells, Rab11a plays a role in autophagosome maturation and fuses with autophagosomes to form amphisomes for virus release (Wang et al., 2017a). In addition, NSP2 colocalizes with LC3 and activates incomplete autophagy to enhance viral replication by inhibiting the fusion of lysosomes and autophagosomes (Sun et al., 2012a). Except for NSP2, NSP3 can also activate the formation of autophagosomes (Zhang et al., 2019b). Furthermore, it has been verified that PRRSV activates mitophagy, lipophagy, and aggrephagy (Li et al., 2016; Wang et al., 2019a; Cao et al., 2020). The infection of PRRSV in Marc145 cells activates mitophagy to promote virus replication (Li et al., 2016), and PRRSV downregulates N-myc downstream regulated 1 (NDRG) to promote lipophagy, thus resulting in the production of large amounts of free fatty acids to provide materials for virus replication (Wang et al., 2019a). In addition, PRRSV infection can induce aggrephagy through the binding of NSP2 tail domain to cellular protein 14-3-3 ϵ (Cao et al., 2020). Notably, the PRRSV-induced autophagy of thymic epithelial cells can regulate the development of T cells (Wang et al., 2020).

Porcine coronaviruses are positive single-strand RNA viruses with an envelope (Gorbalenya et al., 2006; Guo et al., 2016). At present, coronaviruses are divided into four genera according to phylogenetic clustering: alphacoronavirus, betacoronavirus, gammacoronavirus, and deltacoronavirus (Carstens and Ball, 2009; Woo et al., 2010; Guo et al., 2016). Transmissible gastroenteritis virus (TGEV) and porcine epidemic diarrhea virus (PEDV) belong to alphacoronavirus, porcine hemagglutinating encephalomyelitis virus (PHEV) belongs to betacoronavirus, and porcine deltacoronavirus (PDCOV) belongs to deltacoronavirus (de Groot et al., 2011). TGEV causes watery diarrhea, dehydration, and vomiting in two-week-old piglets (Underdahl et al., 1975). PEDV is the pathogen of epidemic diarrhea in pigs, which can cause severe diarrhea and vomiting in sucking piglets with high mortality (Li et al., 2012; Crawford et al., 2016). PHEV infection usually causes encephalomyelitis and vomiting in piglets (Ding et al., 2017), and PDCOV infection causes severe dehydration and vomiting in piglets (Jung et al., 2015; Jung et al., 2016). Many studies have reported an interaction between porcine coronaviruses and autophagy. (1) TGEV and autophagy: At present, there is controversy about the interaction between TGEV and autophagy. Guo et al. (2016) reported that TGEV infection in ST and PK15 cells increases the bilayer and monolayer vesicle structures, and increases esterified LC3. TGEV replication is negatively regulated by autophagy (Guo et al., 2016). However, another research team reported that TGEV infection in porcine epithelial cells (IPEC-J2) induces mitophagy to maintain cell survival and potentially promote virus replication (Zhu et al., 2016). This finding suggests that autophagy induced by TGEV infection in different cells varies and may have multiple mechanisms. (2) PEDV and autophagy: The research findings on the relationship between PEDV and autophagy are also controversial. Vero and IPEC-J2 cells infected with PEDV show autophagy activation, which promotes virus replication (Guo et al., 2017; Lin et al., 2020). PEDV infection can activate ERS, depending on the PERK and IER1 pathways, by increasing ROS production to activate autophagy (Sun et al., 2021). Controversially, Ko et al. (2017) reported that PEDV infection in porcine intestinal epithelial cells (IECs) activates autophagy, which in turn inhibits viral replication. Similarly. PEDV infection in Vero and LLC-PK1 cells upregulates the expression of bone marrow stromal cell antigen 2 (BST2), which recruits the E3 ubiquitin ligase membrane-associated ring-CH-type finger 8 (MARCH8) to catalyze the ubiquitination of PEDV N protein. The ubiquitinated N protein was recognized by NDP52, delivered to autophagy-lysosomes, and selectively degraded, resulting in the inhibition of virus replication (Kong et al., 2020). Several PEDV viral proteins play roles in autophagy activation. Nsp6 activates autophagy mainly through the PI3K/Akt/mTOR pathway, while ORF3 upregulates the GRP78 protein and activates the PERK-eIF2α signal pathway to induce ERS, thus activating autophagy (Zou et al., 2019; Lin et al., 2020). (3) PHEV and autophagy: PHEV infection activates autophagy independent of the classic AMPK-mTORC1-ULK1 pathway (Li et al., 2021c). Additionally, PHEV induces incomplete autophagy, which is necessary for replication (Ding et al., 2017). Mechanismly, PHEV inhibits mTORC1 activation to activate atypical autophagy by downregulating the expression of transcription factor EB (TFEB) (Wang et al., 2021b). (4) PDCOV and autophagy: PDCOV infection activates complete autophagy through the p38 signal pathway to promote virus replication (Qin et al., 2019; Duan et al., 2021). It is worth noting that inactivated PDCOV can also activate autophagy, but it is not as strong as infectious PDCOV (Duan et al., 2021).

### The Role of Autophagy in Viruses Causing Avian Diseases

#### Avian DNA Viruses

Egg drop syndrome virus (EDSV) belongs to the genus *Atadenovirus* of the family *Adenoviridae*, and its genome is double-stranded linear DNA (Fu et al., 2013; Larson et al., 2015). The quality of poultry eggs decreases due to EDSV, and the clinical symptoms are soft-shell eggs, thin-shell eggs, and shell-free eggs (Huang et al., 2015). To date, only one report has verified the interaction between EDSV and autophagy. Wang et al. (2018) reported that EDSV infection in duck embryo fibroblasts (DEFs) cells activates complete autophagy through the class I PI3K/Akt/mTOR pathway and exploits autophagy to enhance its replication.

Duck enteritis virus (DEV) is the pathogen of duck viral enteritis, causing damage to the blood vessels, intestinal mucosa, and lymphoid organs in ducks (Wang et al., 2013). DEV is a member of the family *Herpesviridae*, the subfamily *Alphaherpesvirinae*, and the genus *Mardicirus*. The genome is double-stranded linear DNA (Wu et al., 2019). Growing evidence has shown that DEV can induce autophagy. Yin et al., (2017c) reported that DEV infection activates complete autophagy, which is beneficial for virus replication in duck embryo fibroblast (DEF) cells. Subsequently, it was found that DEV infection could activate autophagy through a variety of mechanisms. First, DEV activates autophagy by activating ERS, mediated by the activation of the PERK and IRE1 pathways (Yin et al., 2017a). Second, DEV induces autophagy through the activation of the AMPK-TSC2-mTOR signaling pathway mediated by energy metabolism damage (Yin et al., 2017b). Third, DEV infection induces autophagy by increasing the concentration of Ca^2+^ in the cytoplasm and activating the CaMKKβ-AMPK signaling pathway (Yin et al., 2018). Fourth, DEV infection downregulates miR-30a-5p, leading to the upregulation of beclin-1 and increasing beclin-1-mediated autophagy (Wu et al., 2019).

#### Avian RNA Viruses

Influenza A viruses (IAVs) are members of the *Orthomyxoviridae* family and contain a segmented single-strand RNA (Zeng et al., 2021). IAVs are important zoonotic pathogens (Zeng et al., 2021). They can be divided into different subtypes according to the glycoprotein hemagglutinin (HA) and neuraminidase (NA) (Hsu, 2018). At present, there are 18 HA subtypes and 11 NA subtypes (Zhang et al., 2019a). The interaction between autophagy and IAVs has been widely studied. Here, we briefly summarize the interactions between H1N1, H9N2, H5N1, and autophagy. (1) H1N1 and autophagy: H1N1 infection in A549 and MEF cells induced incomplete autophagy, which contributes to IAV replication in a time-dependent manner (Feizi et al., 2017; Yeganeh et al., 2018; Zhang et al., 2020a) Mechanismly, H1N1 activates autophagy by activating the PI3 K pathway (Zhang et al., 2019a). H1N1 also downregulates heterodimeric transcription factor hypoxia-inducible factor 1 (HIF-1α) to reduce glycolysis, and subsequently increases autophagy mediated by the AMPKα-ULK1 signaling pathway (Zhao et al., 2020). At the early stage of infection, H1N1 degrades the antioxidant enzyme superoxide dismutase type 1 (SOD1) by autophagy, leading to the production of ROS (Jung et al., 2018). H1N1 NS1 indirectly induces autophagy by upregulating the synthesis of HA and M2 (Zhirnov and Klenk, 2013). Controversially, Yu et al. (2019) found that H1N1 inhibits autophagy by promoting the formation of circular RNA GATAD2A in A549 cells, thereby promoting virus replication. (2) H5N1 and autophagy: At present, it is reported that H5N1 has a variety of mechanisms to activate autophagy and promote virus replication. H5N1 can activate autophagy through the AKT-mTOR or TSC2 pathway (Ma et al., 2011; Sun et al., 2012b). In addition, H5N1 induces functional autophagy by activating JNK and inhibiting the PI3 K pathway (Zhang et al., 2019a). Another study demonstrated that PB2, NP, and M2 encoded by H5N1 cooperatively functioned in autophagy activation. Initially, the interaction between PB2 protein and heat shock protein 90 kDa α [cytosolic], class A member 1 (HSP90AA1) promotes the synthesis of viral RNA. The binding of NP and LC3 is beneficial to the export of vRNP. Subsequently, the interaction between M2 and LC3 leads to the release of infectious viral particles, thus accelerating the production of the virus offspring (Wang et al., 2019b). (2) H9N2 and autophagy: H9N2 induces autophagy by regulating oxidative stress *via* the Akt/TSC2/mTOR pathway, activates autophagy by activating both the PI3K and JNK pathways, and promotes virus replication (Zhang et al., 2021c). In short, autophagy is activated in host cells infected with IAV, with some differences in the upstream pathway according to different subtypes (Zhang et al., 2019a).

NDV is a member of the genus *Avulavirus* of the family *Paramyxoviridae*. Its genome is a single negative-stranded RNA (Alexander, 2000). Birds are the natural hosts of NDV, making it highly contagious in birds. At present, there are many studies on the interaction between NDV and autophagy. Meng et al. (2012a) found that NDV infection in U251 cells activates complete autophagy to promote virus replication (Meng et al., 2012a). It has been verified that NDV infection activates autophagy to maintain cell survival and NDV replication in chicken cells and tissues (Sun et al., 2014; Kang et al., 2017). In addition, the low virulent strain of NDV and its recombinant strain can activate autophagy (Meng et al., 2014; Bu et al., 2015; Bu et al., 2016). The NDV low virulent strain LaSota activates mitophagy to promote virus replication by inhibiting apoptosis (Meng et al., 2014). Moreover, the recombinant avirulent NDV LaSota strain expressing the rabies virus glycoprotein (rL-RVG) activates ERS through three branches of AFT6, PERK, and IRE1, thus activating autophagy in gastric carcinoma cells (Bu et al., 2015; Bu et al., 2016). Notably, NDV-encoded nucleocapsid protein (NP) or phosphoprotein (P) can effectively activate autophagy, and Hemagglutinin-neuraminidase (HN) and fusion (F)proteins cooperatively activate autophagy (Cheng et al., 2016; Ren et al., 2019). HN and F proteins activate autophagy by activating the AMPK/mTORC/ULK1 pathway (Cheng et al., 2016; Ren et al., 2019). Remarkably, NDV infection leads to the reprogramming of cellular energy metabolism *via* the degradation of sirtuin 3 (SIRT3) by mitophagy (Gong et al., 2021).

Infectious bronchitis virus (IBV) is a kind of avian coronavirus that is the pathogen of avian bronchitis and causes great losses to the poultry industry (Cottam et al., 2014). IBV belongs to the subfamily *Coronavirinae*, family *Coronaviridae*, and order *Nidovirales*, and the genome is positive for single-strand RNA (Cottam et al., 2011; Fung and Liu, 2019). Increasing evidence shows that IBV interacts with autophagy. Cottam et al. (2011) found that IBV-encoded nonstructural protein 6 (nsp6) promoted the formation of autophagosomes and fusion with lysosomes through the activation of class III PI3K. In addition, nsp6 inhibits the expansion of autophagosomes (Cottam et al., 2014). Moreover, ATG5, IRE1, and ERK1/2 are necessary for autophagy induced by IBV infection (Fung and Liu, 2019).

Infectious bursal disease virus (IBDV) is the pathogen causing bursa of Fabricius injury in birds, especially in 3–6-week-old chickens with high mortality (Burkhardt and Muller, 1987; Sharma et al., 2000). IBDV belongs to the genus *Avibirnavirus* in the family *Birnaviridae* (Muller et al., 1979). The genome is segment double-stranded RNA (dsRNA) (Muller et al., 1979; Li and Zheng, 2020). Evidence suggests that IBDV can subvert autophagy to promote virus replication. At the early stage of IBDV infection, LC3-II flux and dephosphorylation of AKT-mTORC increases (Hu et al., 2015). VP2 binds to HSP90AA1 to trigger autophagy through the AKT-mTORC pathway, and knockdown of HSP90AA1 inhibits autophagy (Hu et al., 2015). Additionally, at the early stages of IBDV infection, VP3, another viral protein, binds to beclin-1 and 3-phosphoinositide dependent protein kinase 1 (PDRK1), destroying the PIK3C3-beclin-1 and PIK3C3-PDPK1 complexes, and inhibiting the formation and maturation of autophagosomes (Zhang et al., 2020b). At the late stage of infection, IBDV increases the flux of LC3-II and promotes the fusion of autophagosomes and lysosomes, but p62 is not degraded (Wang et al., 2017c). Transmission electron microscopy showed that the intact IBDV virions were arranged around p62, indicating that virus-induced incomplete autophagy provides an acid environment for virus maturation and release (Wang et al., 2017c).

Avian reovirus (ARV) and Muscovy duck reovirus (MDRV) belong to the genus *Orthoreovirus* of the family *Reoviridae*, and their genomes are segmented into dsRNA (Hieronymus et al., 1983). ARV is the pathogen of viral arthritis, chronic respiratory disease, egg drop, dwarf syndrome, and malabsorption syndrome in chickens, while MDRV is the pathogen of liver white spot disease in ducklings (Benavente and Martinez-Costas, 2007; Wu et al., 2017). Both ARV and MDRV activate autophagy and exploit autophagy to promote self-replication. ARV infection in chicken cells induces complete autophagy through the PI3K/Akt/mTOR pathway to assist virus replication (Meng et al., 2012b; Duan et al., 2015). The NLS region of the nonstructural protein p17 encoded by ARV induces autophagy by activating phosphatase and tensin deleted on chromosome 10 (PTEN), AMPK, and dsRNA-dependent protein kinase (PKR)/eIF2 pathways (Chi et al., 2013; Li et al., 2015). Moreover, p17 mediates the inhibition of Akt, leading to the activation of autophagy (Huang et al., 2017). In comparison, MDRV infection activates incomplete autophagy (Li et al., 2021b). MDRV promotes the fusion of autophagosomes and lysosomes, but inhibits the degradation of autolysosomes. The envelope protein σA and nonstructural protein σNS encoded by MDRV play important roles in this process (Wu et al., 2017; Li et al., 2021b).

ALV-J belongs to the genus of *Alpharetrovirus* of the *Retroviridae* family, and its genome is RNA (Rajabzadeh et al., 2010). ALV-J is a pathogen that causes tumor-related diseases in poultry (Lu et al., 2013). At present, few studies have examined the interaction between ALV-J and autophagy. Lu et al. (2013) reported for the first time that ALV-J infection in DF-1 cells decreases the expression of LC3-II and autophagosome-related proteins, and autophagy activated by rapamycin treatment inhibits virus replication, suggesting that ALV-J infection enhances virus replication by inhibiting autophagy. Another study demonstrated that ALV-J inhibits autophagy through the GADD45β/MEKK4/p38MAPK signaling pathway (Liao et al., 2020).

The Duck hepatitis A virus (DHAV) is a member of the genus *Avihepadnavirus* of the family *Picornaviridae*, and the viral genome is a positive single-stranded RNA (Liu et al., 2021). DHAV has a high mortality rate in young ducklings (Ming et al., 2019). DHAV infection in duck embryonic hepatocytes (DEHs) cells increases incomplete autophagy and promotes virus replication (Ming et al., 2019). The viroporin-like 2B protein encoded by DHAV-1 has been proven to induce incomplete autophagy (Liu et al., 2021).

### The Role of Autophagy in Viruses Causing Ruminant Diseases

Bluetongue virus (BTV) is a member of the genus *Orbivirus* in the family *Reoviridae*, and the genome is dsRNA (Lv et al., 2015b). BTV is a pathogen of bluetongue in wild and domestic ruminants (Maclachlan, 2011). The replication of BTV1 activates complete autophagy to promote replication (Lv et al., 2015b). Subsequently, three upstream pathways were verified in the process of BTV-induced autophagy: (1) BTV contributes to the initiation of autophagy by inhibition of the AKT-TSC2-mTOR pathway and activation of the AMPK-TSC2-mTOR pathway (Lv et al., 2016b), (2) BTV activates autophagy by ERS mediated by the PERK-eIF2β pathway (Lv et al., 2015a), and (3) BTV activates autophagy by destroying cell energy metabolism (Lv et al., 2016a).

Peste des petits ruminants virus (PPRV) is a pathogen that causes mummified fetus, miscarriage, and lamb death in pregnant ewes (Borel et al., 2005; Abubakar et al., 2008). PPRV belongs to the genus *Morbillivirus* in the family *Paramyxoviridae*, and the genome is a single negative-strand RNA (Yang et al., 2018). Accumulating studies have shown an interaction between autophagy and PPRV. PPRV infection in caprine endometrial epithelial cells (EECs) activates complete autophagy and may be mediated by nonstructural protein C and nuclear protein N. PPRV exploits autophagy to promote replication (Yang et al., 2018). Autophagy activation leads to the inhibition of caspase-dependent apoptosis, thus promoting PPRV infection (Yang et al., 2018). Strikingly, attenuated PPRV strains induce two sets of autophagy flux at 1.5 h and 9-24 h post infection, respectively (Yang et al., 2020a). At the early stage of infection, the interaction between the PPRV H protein and nectin cell adhesion molecule 4 (NECTIN4) inhibits the activity of the AKT-MTOR pathway and triggers autophagy (Yang et al., 2020a). At the late stage of infection, C protein and N protein encoded by PPRV bind to IRGM and heat shock 70 kDa protein 1A (HSPA1A), respectively, to induce autophagy (Yang et al., 2020a).

Caprine parainfluenza virus type 3 (CPIV3) is a pathogen that causes severe respiratory diseases in goats (Li et al., 2014b). It belongs to the genus *Respirovirus* of the family *Paramyxiviridae*, and its genome is a single negative-stranded RNA (Li et al., 2020). To date, only one report has shown that CPIV3 exploits exosomes-mediated autophagy inhibition to maintain virus replication in MDBK cells (Mao et al., 2020).

Epizootic hemorrhagic disease virus (EHDV) is a member of the genus *orbiviruses* of the *Reoviridae* family, and its genome is a double-stranded segment of RNA (Shai et al., 2013). EHDV infection usually results in the death of white-tailed deer (Shai et al., 2013), and with spontaneously immortalized ovine kidney (OK) cells, EHDV infection activates autophagy mediated by activation of the JNK pathway and exploits autophagy to promote virus replication (Shai et al., 2013).

Bovine viral diarrhea virus (BVDV) is a member of the genus *Pestivirus* (*Flaviridae*), and its genome is single positive-stranded RNA (Fu et al., 2014a). BVDV is a pathogen that causes serious damage to cattle productivity and has a high fatality rate for cattle (Perdrizet et al., 1987). Both cytopathic and non-cytopathic BVDV can induce autophagy to promote virus replication (Rajput et al., 2017). BVDV-encoded glycoprotein Erns, E2, and nonstructural protein NS4B are involved in the induction of autophagy (Fu et al., 2014b; Suda et al., 2019).

Bovine epidemic fever virus (BEFV), also known as three-day sickness or three-day fever, is a pathogen that causes a decline in milk production in dairy cattle and in the quality of beef cattle (Akakpo, 2015). BEFV belongs to the genus *Ephemerovirus* within the family *Rhabdoviridae*, and the genome is single negative-sense RNA (Lee, 2019; Tseng et al., 2020). BEFV infection in MDBK cells induces autophagy by upregulating the PI3K/Akt/NF-κB and Src/JNK/AP1 pathway at the early and middle stages of infection. At the late stage of infection, autophagy is activated by inhibition of the PI3K/Akt/mTOR pathway, which is mediated by the M protein encoded by BEFV (Cheng et al., 2019). The activation of autophagy promotes virus replication (Cheng et al., 2019).

### Other Animal Viruses and Autophagy

Rhesus monkey rhadinovirus (RRV) is a member of gamma *herpesvirus*, which causes primary effusion lymphoma (PEL) or body cavity-based lymphoma (BCBL), and multicentric Castleman’s disease, which is closely related to KSHV (Desrosiers et al., 1997; Ritthipichai et al., 2012). During latency, the vFLIP protein encoded by RRV maintains cell survival by activating autophagy and inhibiting apoptosis (Ritthipichai et al., 2012).

EHV-1 is a pathogen that causes respiratory diseases, abortion, and neurological disorders in horses. EHV-1 belongs to the *Alpaherpesvirinae* subfamily and has a double-stranded DNA genome (Cymerys et al., 2014). EHV-1 infection in the primary culture of murine neurons induces autophagy (Cymerys et al., 2014). However, EHV-1-induced autophagy has no effect on EHV-1 replication (Cymerys et al., 2014).

Rabbit hemorrhagic disease virus (RHDV) is a member of the *Cycloviridae* family that contains a single positive-stranded RNA genome (Vallejo et al., 2014). RHDV is a pathogen that causes an acute and highly fatal disease in wild and domestic rabbits (Vallejo et al., 2014), causing acute liver failure (Lee, 2012). RHDV infection of liver cells increases the flux of LC3-II and the degradation of p62, indicating that RHDV induces complete autophagy (San-Miguel et al., 2014). RHDV also activates mitochondrial autophagy to promote virus replication (San-Miguel et al., 2014; Crespo et al., 2020). Controversially, Vallejo et al. (2014) reported that autophagy is rapidly activated through ERS at the early stage of RHDV infection to protect liver cells, while at the late stage of infection, RHDV promotes apoptosis to inhibit autophagy, thereby increasing virus replication.

Mouse herpesviruses include murine cytomegalovirus (MCMV) and murine gammaherpesvirus 68 (MHV68), and their genomes are DNA. MCMV belongs to the *β herpesvirus* subfamily and is a pathogen that causes malignant tumors or low immune function (Chaumorcel et al., 2008). MHV68 is a member of the *γ herpesvirus* subfamily and is the pathogen causing lymphoid and epithelial tumors in animals (Cuconati and White, 2002). In a mouse model of MCMV infection, MCMV activates autophagy through the mTOR signal pathway, protecting the survival of retinal cells (Mo et al., 2019). MCMV infection also promotes the formation of autophagosomes at the early stage of infection, while inhibiting autophagy at the late stage of infection. Autophagy is beneficial for virus replication (Mo et al., 2014). Meanwhile, Zhang et al. reported that MCMV inhibits the autophagy process to reduce virus release at the early stage of infection, while at the late stage of infection, decreased virus replication is mainly dependent on apoptosis (Zhang et al., 2021d). During the latent period, MHV68 inhibits autophagy through the binding of the virus M11 protein to beclin-1 (Ku et al., 2008; Su et al., 2014). The activation of autophagy contributes to the latent reactivation of MHV68 (Su et al., 2014).

### Autophagy Crosstalk With Innate Immunity Under Animal Virus Infection

Mounting studies have shown that autophagy has a protective effect against pathogens; it can activate the immune response to play an antiviral effect. However, some viruses have evolved to destroy or use autophagy mechanisms to evade the host’s immune response. Accumulating studies have confirmed the interaction between autophagy and innate immunity under virus infection. In this chapter, we summarize how autophagy regulates the immune response under the condition of an animal virus infection.

Animal virus infection uses autophagy to activate or inhibit the innate immune response. On the one hand, the activation of autophagy triggers an immune response for antiviral effects. For example, PPRV infection activates the expression of ATG13, which increases interferon (IFN)-β production mediated by retinoic acid-inducible gene I (RIG-I) and the expression of cytokines, thus inhibiting the replication of PPRV (Ma et al., 2020). On the other hand, the virus exploits autophagy to inhibit the innate immune response and promote its own replication, such as FMDV, ASFV, JEV, AIVs, and BTV. During FMDV infection, overexpressed ATG5-ATG12 activates the TANK binding kinase 1 (TBK1)-interferon regulatory factor 3 (IRF3)-mediated NF-κB pathway by inhibiting TRAF3 degradation. In response, the FMDV 3C protein degrades ATG5-ATG12 to inhibit antiviral innate immunity (Fan et al., 2017). In addition, FMDV 3A protein degrades Ras-GTPase-activating protein (GAP)-binding protein 1 (G3BP1) by upregulating the expression of autophagy-related gene leucine-rich repeats containing 25 (LRRC25), thus inhibiting the expression of type I IFN (Yang et al., 2020b). ASFV activates autophagy through MGF-505-7R to degrade the antiviral gene stimulator of interferon response cGAMP interactor 1 (STING), thus increasing virus replication (Li et al., 2021a). In addition, JEV can inhibit the expression of mitochondrial antiviral signaling protein (MAVS) and IRF3 by activating autophagy (Jin et al., 2013). Zeng et al. (2021) reported that the PB1 protein of the H7N9 virus promotes E3 ligase ring finger protein 5 (RNF5) to catalyze the K27-linked polyubiquitination of MAVS at Lys362 and Lys461, and facilitates NBR1 to recognize MAVS and transport MAVS to autolysosomes for degradation. BTV exploits autophagy to degrade the signal transducer and activator of transcription 2 (STAT2), thus blocking the downstream signaling pathway of IFN-I (Avia et al., 2019).

## Concluding Remarks

Increasing evidence of the role of autophagy in animal virus replication and pathogenesis suggests that the modulation of autophagy may represent a novel therapeutic strategy against virus infection. In this review, we summarized the virus regulation of autophagy, the effect of autophagy on virus replication, and the mechanisms involved in virus-autophagy interaction. In conclusion, it becomes clear that most animal viruses, including porcine and avian viruses, induce autophagy to promote virus replication, suggesting that during long-term coexistence, viruses evolve in various ways to exploit host autophagy. In comparison, a few viruses, such as PRV, PCVs, TGEV, and ALV-J, inhibit autophagy in specific cell lines. Notably, virus-induced autophagy may be affected by different cell types, viral doses, virus strains, and infectious time points, which may explain the controversial consequences of virus-autophagy interactions under different circumstances.

Although the interaction between autophagy pathways and animal viruses has been widely reported, there are still many deficiencies. For example, most of the results were still preliminary. There is still much to learn about the basic mechanisms of how animal viruses exploit autophagy and how animals use the machinery to protect themselves. In addition, autophagy has been proven to have wide crosstalk with various important cellular biological processes, such as immunity, metabolism, and cell death. How autophagy influences these biological processes and whether these processes play a role in response to animal viruses need to be further elucidated. More importantly, compared with studies on human viruses and autophagy, it is easier to carry out *in vivo* experiments in animals to elucidate the mechanism of animal virus and autophagy interactions more precisely. Nevertheless, only a few studies have conducted *in vivo* experiments, which limits the application of virus-autophagy theory in antiviral therapeutics and vaccine development.

Accumulating evidence demonstrated that autophagy promotes the replication of most animal viruses, that is to say, autophagy inhibition can be used as a potential therapeutic strategy to protect animals. Therefore, we propose the following three possible applications: (1) The administration of autophagy inhibitors, such as CQ and 3-MA, to help rescuing some specific animals like breeding stock, specialty animals or pets. (2) The adjunction of fatty acids or glucose to animal feed to activate mTORC1, thereby inhibiting autophagy in animals. (3) The usage of autophagy inhibitor as vaccine adjuvants in combination with vaccines for better protection.

It is noteworthy that for most livestock, the drug treatment for infected animals is too expensive to be worth it. Since autophagy is induced upon various cellular stress events, such as starvation, endoplasmic reticulum stress and oxidative stress. Moreover, it is understood that more than 90% of animal virus-infected host cells can induce and exploit autophagy to promote their own replication. Therefore, under certain circumstances, such as vaccine inoculation, livestock transportation, etc., it is feasible to prevent infectious disease by modulation of autophagy to alleviate the damage of livestock. In another way, unlike human beings, the production performance is an important index to evaluate the value of the animals. Under physiological conditions, autophagy regulates animal reproduction, growth, immune system and meat maturation. Whether autophagy is beneficial or detrimental depends on the appropriate duration and degree of induction. For example, in laying period, autophagy is involved in the regression of ruptured follicles after ovulation, especially in the egg membrane layer (Tesseraud et al., 2021). Under the situation like this, other impact of autophagy should be taken into account when using autophagy inhibitors.

In conclusion, recent studies have demonstrated that there is more diversity in the role of autophagy than was hitherto appreciated. The typical example is the regulation of innate immunity and energy metabolism by autophagy reported recently. The application of autophagy may become a potentially important strategy to combat especially those intractable animal viral infectious diseases. Understanding the interaction between animal viruses and autophagy provides a theoretical basis for effective antiviral therapy.

## Author Contributions

YS contributed to the conception of the study. HJ wrote the first draft of the manuscript. XK wrote sections and drew the figures of the manuscript. YS and CD performed the final corrections. All authors contributed to the manuscript revision and approved the submitted version.

## Funding

This work was funded by grants 32122085 (to YS) and 31872453 (to YS) from the National Natural Science Foundation of China. The funders had no role in study design, data collection and interpretation, or the decision to submit the work for publication.

## Conflict of Interest

The authors declare that the research was conducted in the absence of any commercial or financial relationships that could be construed as a potential conflict of interest.

## Publisher’s Note

All claims expressed in this article are solely those of the authors and do not necessarily represent those of their affiliated organizations, or those of the publisher, the editors and the reviewers. Any product that may be evaluated in this article, or claim that may be made by its manufacturer, is not guaranteed or endorsed by the publisher.
